# Selective Oxidation of Halophenols Catalyzed by an Artificial Miniaturized Peroxidase

**DOI:** 10.3390/ijms24098058

**Published:** 2023-04-29

**Authors:** Daniele D’Alonzo, Maria De Fenza, Vincenzo Pavone, Angela Lombardi, Flavia Nastri

**Affiliations:** Department of Chemical Sciences, University of Napoli Federico II, Via Cintia, 80126 Napoli, Italy; dandalonzo@unina.it (D.D.); maria.defenza@unina.it (M.D.F.); vincenzo.pavone@unina.it (V.P.); alombard@unina.it (A.L.)

**Keywords:** artificial peroxidase, halophenols, miniaturization, metalloenzyme design, chemoselectivity

## Abstract

The development of artificial enzymes for application in sustainable technologies, such as the transformation of environmental pollutants or biomass, is one of the most challenging goals in metalloenzyme design. In this work, we describe the oxidation of mono-, di-, tri- and penta-halogenated phenols catalyzed by the artificial metalloenzyme Fe-MC6*a. It promoted the dehalogenation of 4-fluorophenol into the corresponding 1,4-benzoquinone, while under the same experimental conditions, 4-chloro, 4-bromo and 4-iodophenol were selectively converted into higher molecular weight compounds. Analysis of the 4-chlorophenol oxidation products clarified that oligomers based on C-O bonds were exclusively formed in this case. All results show that Fe-MC6*a holds intriguing enzymatic properties, as it catalyzes halophenol oxidation with substrate-dependent chemoselectivity.

## 1. Introduction

Metalloenzymes are very important tools in catalysis, thanks to their huge functional diversity, unrivaled efficiency and high level of selectivity [1,2,3,4]. Given their evolution-driven ability to activate even inert molecules and to produce high-added-value chemicals under mild conditions, metalloenzymes are not only essential for metabolic functions, but they also have a role in a variety of research areas, including asymmetric synthesis, biosensing, synthetic biology and industrial microbiology [5]. The outstanding catalytic properties of metalloenzymes are the result of a complex and strong interplay between metal cofactors and protein scaffolds, mutually affecting enzymatic function and substrate selectivity [1,6]. Indeed, the protein matrix tunes the reactivity of metal ions through first and second sphere interactions while influencing substrate access owing to its inherently chiral environment [7]. Furthermore, in many cases the metal cofactor assists in protein folding, contributing to making the whole molecule catalytically functional [8].

Several metalloenzymes are, however, innately characterized by narrow substrate selectivity, as natural selection resulted in unique binding elements for specific enzymatic functions. The intricate network of non-covalent interactions necessary for protein folding and activity makes metalloenzymes highly sensitive to experimental reaction conditions [6]. After decades of efforts aimed at overcoming these limitations, researchers have now impressively enriched the catalytic toolbox by constructing artificial metalloenzymes [9,10]. Whether exploiting one of the numerous strategies [11,12], including but not limited to miniaturization [13], de novo design [14,15,16,17,18], rational design [19,20], directed evolution [21,22] or supramolecular assembly [23], minimal or more extensive changes to the protein scaffold and/or the metal cofactor have been conceived, enabling the development of new-to-nature architectures with enhanced features compared with native metalloenzymes. Several examples of designed and engineered metalloenzymes endowed with higher catalytic robustness, such as stability under operating conditions and tolerance to non-aqueous solvents [24,25,26], a broader substrate specificity (even toward non-natural catalysis [27,28]) and more effective substrate selectivity [9,14] have been reported.

**Figure 1:**
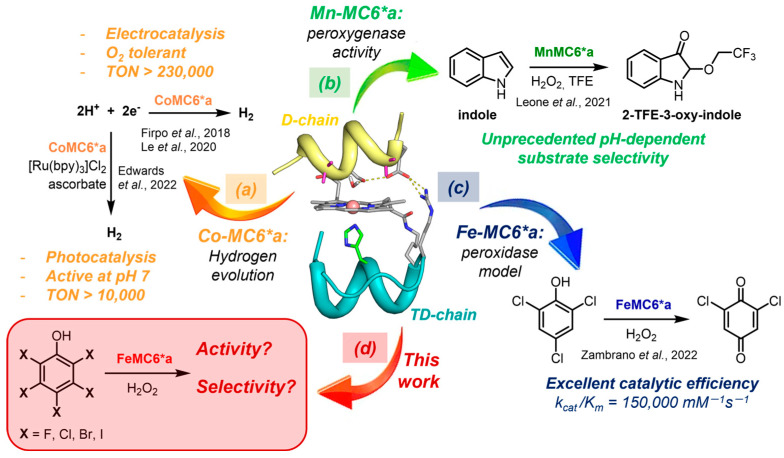
Molecular model of the MC6*a scaffold, with indications of its catalytic potential. The two peptide chains are depicted in different colors: tetradecapeptide (TD) in cyano and decapeptide (D) in yellow. The corresponding sequences are: TD chain, Ac-D-L-Q-Q-L-H-S-Q-K-R-K-I-T-L-NH_2_; D-chain, Ac-D-E-U-Q-L-S-U-Q-K-R-NH_2_. (**a**) Catalytic properties of Co-MC6*a in hydrogen evolution reaction [31,32,33]; (**b**) peroxygenase activity of Mn-MC6*a in indole oxidation [35]; (**c**) catalytic potential of Fe-MC6*a in TCP oxidation [38]; (**d**) this work: evaluation of the activity and selectivity of Fe-MC6*a in the oxidation of variously halogenated phenols. Adapted with permission from Ref. [11], 2019, Elsevier.

In this respect, an illustrative example has been provided by our long-studied family of miniaturized metalloenzymes called “mimochromes” (MCs) [4,12,13,29]. MCs havebeen demonstrated to combine remarkable stability, high catalytic potential and tunable enzymatic activity in a single molecule [4,11,29] by virtue of their minimal but finely designed peptide scaffold, involving two alpha-helical peptides covalently linked to a deuteroporphyrin core [30]. The latest and most promising member of this family, MC6*a, is an excellent and versatile catalyst. Its enzymatic activity significantly depends on the nature of the metal ion (Figure 1). The metalloenzyme housing cobalt ion into the porphyrin (Co-MC6*a) acts as a promising catalyst for hydrogen-evolution reactions either in electrocatalytic or photochemical systems [31,32,33] (Figure 1a). The manganese derivative of MC6*a (Mn-MC6*a) represents an efficient peroxygenase, performing highly selective oxidative transformations, such as the sulfoxidation of aryl thioethers [34]. Furthermore, in the model reaction of indole oxidation, Mn-MC6*a is able to oxidize the substrate with unprecedented pH-dependent regioselectivity, providing unnatural chemicals with high synthetic utility [35] (Figure 1b). The iron-containing derivative, Fe-MC6*a, mainly acts as a peroxidase model, with intriguing catalytic properties in the oxidation of various substrates [36,37]. When assayed in the dehalogenation reaction of the pollutant 2,4,6-trichlorophenol (TCP), Fe-MC6*a exhibited the highest catalytic efficiency among both natural and artificial metalloenzymes, tested in the same reaction [38] (Figure 1c).

**Figure 2:**
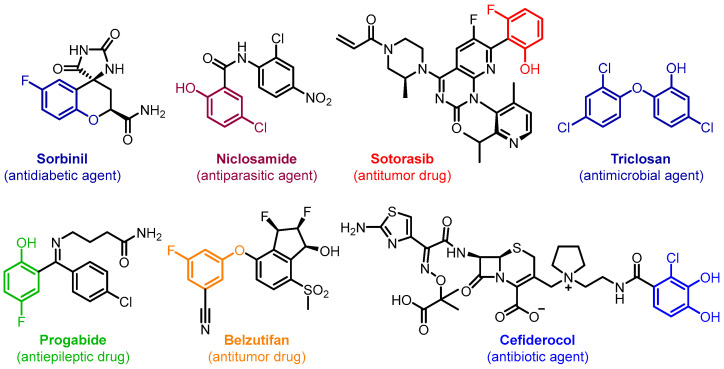
Representative examples of pharmaceuticals containing halophenols and their derivatives. Halophenol moieties are highlighted in different colors.

The excellent behavior of Fe-MC6*a in TCP dehalogenation [38] encouraged us to carry out a more extensive evaluation of its reactivity toward a variety of other halogenated phenols, including mono-, di-, tri- and penta-halophenols (Figure 1d). Halophenols represent interesting substrates for oxidative catalysis. Due to their well-known toxicity [39], their biocatalytic degradation is a highly demanding task in view of prospective applications in environmental biotechnologies [20]. Furthermore, several halophenols represent key components in many pharmaceuticals, and they are used in diverse therapeutic areas [40] (Figure 2). Thus, their selective conversion into higher reactivity synthons may result in being very useful for drug synthesis.

A plethora of biocatalytic halophenol oxidation reactions by both natural and artificial heme-enzymes has been reported in the literature, involving peroxidases [41], globins [42], globin-like enzymes [43] and oxygenases [44]. However, in many cases, low or no selectivity has been associated to these reactions [45,46,47], thus making these biocatalysts of limited interest from a synthetic standpoint. Herein, we report that Fe-MC6*a promotes the efficient oxidation of various halophenols while enabling highly selective transformations, whose fate depends on the nature of the halogen atom. The intriguing enzymatic behavior of Fe-MC6*a further broadens the catalytic potential of this artificial metalloenzyme to produce synthetically useful building blocks and the degradation of organic pollutants.

## 2. Results and Discussion

### 2.1. H_2_O_2_-Dependent Dehalogenation Activity of Fe(III)-MC6*a

The catalytic activity of Fe-MC6*a toward the H_2_O_2_-dependent dehalogenation was first screened on differently substituted halophenols, including mono-, di-, tri- and penta-halophenols, by evaluating the turnover number (TON) [48]. All reactions were performed using the same procedure, with small changes depending on the nature of the substrate. Under typical reaction conditions, H_2_O_2_ was added to a stirring mixture of the given halophenol and Fe-MC6*a, dissolved in a 50 mM phosphate buffer (pH 6.5) solution containing 2,2,2-trifluoroethanol (TFE) as a cosolvent (50% *v*/*v*). After 30 min stirring at room temperature, the reaction was quenched and the mixture analyzed by GC-MS. TON values were determined by measuring the conversion degree based on the substrate consumption, compared to the peak area associated to methyl phenyl sulfone (MPS), used as an internal standard (Table 1).

The position of halogen did not affect TON values. As an example, TONs for the Fe-MC6*a-catalyzed oxidations of 2-chlorophenol (2-CP) and 4-chlorophenol (4-CP) were reasonably similar, as they varied between 310 (2-CP oxidation; entry 1) and 325 (4-CP oxidation; entry 3). Furthermore, the conversion degrees were slightly influenced by the nature of the halogen atom in the substrate, with a progressive decrease in the TON values when the oxidation reaction involved 4-fluorophenol (4-FP, 503 TONs; entry 2), 4-CP (325 TONs; entry 3), 4-bromophenol (4-BP, 235 TONs; entry 4) and 4-iodophenol (4-IP, 208 TONs; entry 5) as substrates. In contrast, the TONs varied dramatically along with the substrate halogenation degree. Indeed, the TON value that referred to the Fe-MC6*a-catalyzed conversion of 4-CP was 325 (entry 3), whereas the reaction with 2,4-dichlorophenol (2,4-DCP) proceeded with a TON value of 2588 (entry 6). As already reported [38], in the case of 2,4,6-trichlorophenol (2,4,6-TCP), the TON value further increased up to 3850 (entry 8). Similarly, the TON that referred to the reaction of 2,4,6-trifluorophenol (2,4,6-TFP) was 3825 (entry 7). Conversely, the oxidations of 2,3,4,5,6-pentafluorophenol (PFP) and 2,3,4,5,6-pentachlorophenol (PCP) were associated with TON values of only 142 and 186, respectively (entries 9 and 10). The observed low TONs for these two substrates could be due to their steric hindrance when approaching the catalytically active Compound I (Cpd I).

### 2.2. Outcome of the Reaction: Case Study of 4-Halophenols

A deep investigation of the nature of the conversion products and their distribution was then carried out. To this end, the Fe-MC6*a-catalyzed dehalogenation reaction involving 4-halophenols was selected as a case study.

#### 2.2.1. Benzoquinone Formation in 4-Fluorophenol Conversion

**Figure 3:**
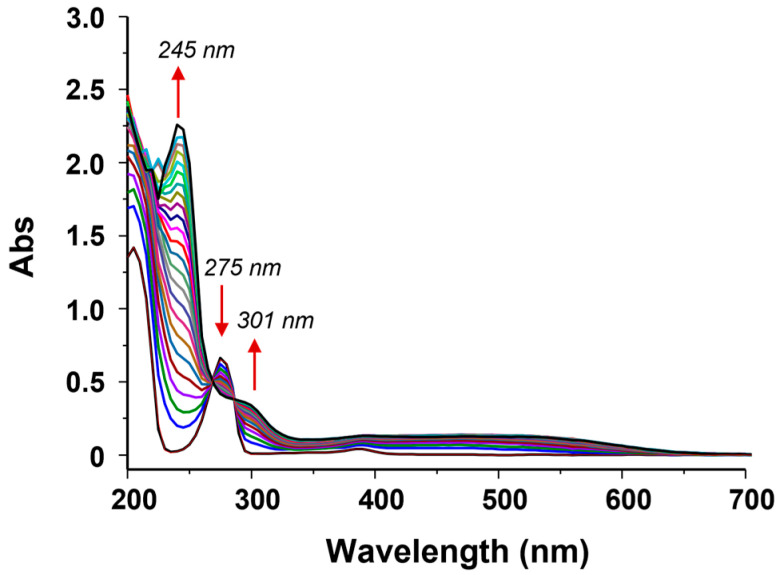
Time-dependent UV–Vis spectra (acquired over 100 min) of Fe(III)-MC6*a (6.25 × 10^−4^ mM) in the presence of 4-FP (0.25 mM) in 50 mM sodium phosphate (pH 6.5) with 50% TFE (*v*/*v*) upon addition of H_2_O_2_ (0.25 mM). Brown line, t = 0 min; black line, 100 min; spectra taken every 5 min are reported in different colors.

The reaction of Fe-MC6*a with H_2_O_2_ and 4-FP was monitored by UV–Vis spectroscopy (Figure 3). Upon H_2_O_2_ addition, a rapid decrease in the 4-FP absorption maximum (275 nm) was observed (Figure 3), while a concurrent increase of bands at 245 and 301 nm, characteristic of 1,4-benzoquinone (BQ), was detected. No reaction progress over the same timescales was observed in the absence of the catalyst.

**Figure 4:**
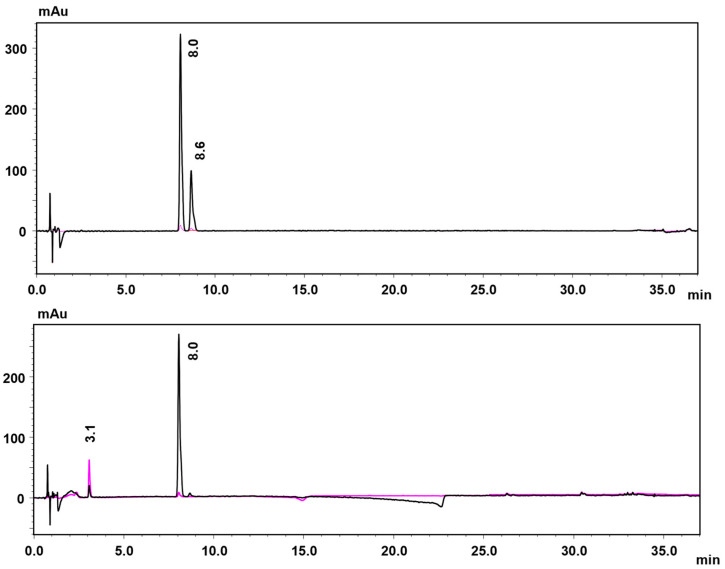
UHPLC traces (black line: λ = 220 nm, pink line: λ = 240 nm) of the Fe-MC6*a-catalyzed oxidation of 4-FP before (upper trace) and 30 min after (lower trace) H_2_O_2_ addition. Reaction conditions: [Fe-MC6*a] = 6.25 × 10^−3^ mM; [4-FP] = 2.5 mM; [H_2_O_2_] = 2.5 mM; phosphate buffer (50 mM) at pH 6.5 with 50% TFE (*v*/*v*). The peak with Rt = 8.0 min is MPS, used as internal standard.

Analysis of the crude mixture for the catalyzed reaction by UHPLC confirmed the formation of a single reaction product (Figure 4).

**Scheme 1:**
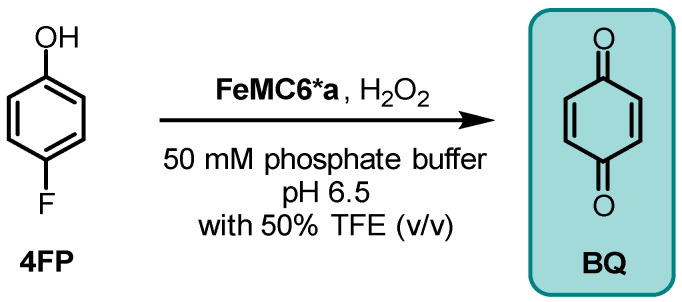
Oxidative defluorination of 4-FP.

Within 30 min of the reaction, a complete conversion of 4-FP (Rt = 8.6 min) into a single oxidation product (Rt = 3.1 min) was observed (Figure 4). The identification of the reaction product was accomplished after extraction of the reaction mixture. A GC-MS analysis of the extracted sample confirmed the formation of BQ as the main dehalogenation product (Scheme 1) because of the presence of a main peak at *m*/*z* 108 ([M]^+^; see Figure S1). Further confirmation of the nature of the oxidation product was provided by analyzing a commercial sample of BQ under the same chromatographic conditions.

It is worth underlining that the outcome of this reaction was significantly different from those observed with most heme-containing metalloenzymes, assayed in the same reaction. Horseradish peroxidase (HRP) and Chloroperoxidase from *Caldariomyces fumago* (CCPO) are reported to provide, along with variable amounts of BQ, coupling products based on unsymmetrical pairing of 4-fluorophenol radicals [46,47]. Low catalytic activity was documented for dehaloperoxidase from *Amphitrite ornata* (DHP-A), as both 4-FP and all the remaining 4-halophenols act as inhibitors based on internal binding [20,49,50,51], 4-FP being the weakest binder of the series. On the other hand, the aerobic oxidation of 4-FP catalyzed by cytochrome P450 (from rat liver microsome) [52] and its mutants [53] provided 1,4-hydroquinone (HQ) as the reaction product, resulting from the reduction of BQ by NADPH-cytochrome P450 reductase. Our result is rather in line with the H_2_O_2_-mediated oxidation reaction performed by another mini-enzyme, the cytochrome c-derived microperoxidase Fe^III^-MP-8, which also converted 4-FP (as well as all other 4-halophenols) into benzoquinone [54] with comparable selectivity.

#### 2.2.2. Coupling Products in 4-Chlorophenol Conversion

**Figure 5:**
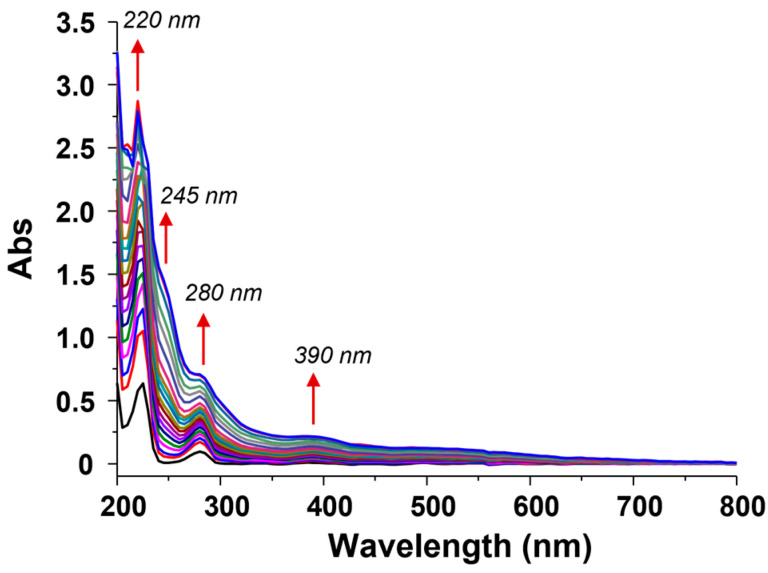
Time-dependent UV–Vis spectra (acquired over 100 min) of Fe(III)-MC6*a (6.25 × 10^−4^ mM) in the presence of 4CP (0.25 mM) in 50 mM sodium phosphate (pH 6.5) with 50% TFE (*v*/*v*) upon H_2_O_2_ addition (0.25 mM). Black line, t = 0 min; red line, 100 min; spectra taken every 5 min are reported in different colors.

The analysis of the reaction products for the Fe-MC6*-catalyzed oxidation of 4-CP turned out to be more challenging. Differently from 4-FP, the UV–Vis spectroscopic analysis of the reaction mixture using 4-CP as a substrate displayed a substantial increase in the intensity of all absorption bands of the starting material (Figure 5), likely suggesting the formation of products structurally similar to the reagent.

The ^1^H NMR analysis of the reaction mixture ([Fe-MC6*a] = 6.25 × 10^−3^ mM, [4-CP] = 2.5 mM, and [H_2_O_2_] = 2.5 mM) (Figure S2) showed a substantial decrease in the intensity of the signals referring to 4-CP (doublets at 6.79 and 7.16 ppm) and a concurrent formation of one main signal at 6.76 ppm, related to BQ, whose intensity was, however, only approximately 5% compared to the starting 4-CP. This finding, together with the observed opalescence of the reaction mixture, suggests the formation of insoluble reaction products.

**Figure 6:**
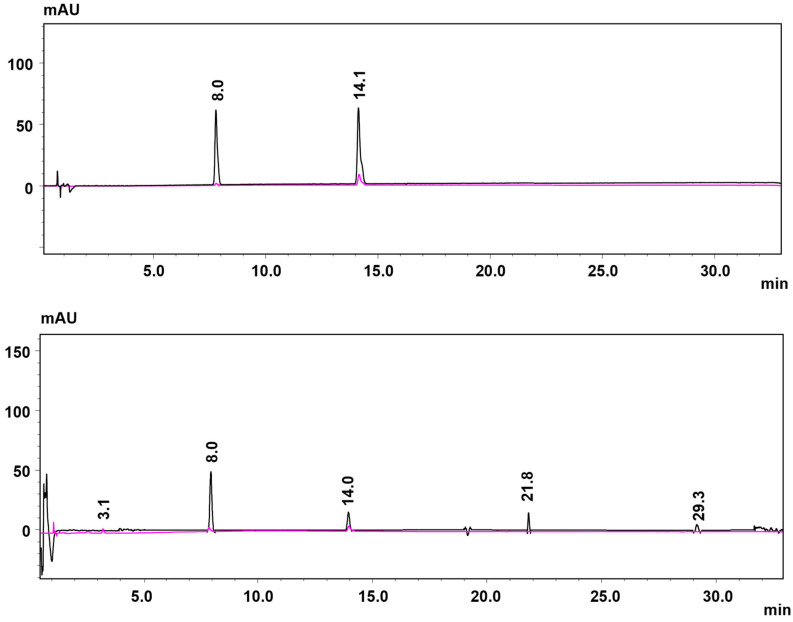
UHPLC traces (black line: λ = 220 nm; pink line: λ = 240 nm) of the Fe-MC6*a-catalyzed oxidation of 4-CP before (upper trace) and 30 min after (lower trace) H_2_O_2_ addition. *Reaction conditions*: [Fe-MC6*a] = 1.0 × 10^−3^ mM; [4-CP] = 2.5 mM; [H_2_O_2_] = 2.5 mM; [MPS] = 2.5 mM; phosphate buffer (50 mM) at pH 6.5 with 50% TFE (*v*/*v*). MPS (peak at Rt = 8.0 min) was used as an internal standard.

Upon removal of the insoluble portion by filtration, an analysis of the reaction mixture by UHPLC after 30 min from H_2_O_2_ addition indicated the conversion of 4-CP (Rt = 14.1 min, Figure 6, upper trace) into a very low amount of BQ (Rt = 3.1 min, Figure 6 lower trace). In contrast, two products with higher retention times with respect to 4-CP (Rt = 21.8 min and 29.3 min, Figure 6 lower trace) were observed.

The UV–Vis spectral profile of the two main peaks related to 4-CP oxidation are similar to 4-CP (Figure S3), showing absorption maxima at λ values (λ_1_ = 211–212 nm, λ_2_ = 293–295 nm) close to those reported for 4-CP (λ = 226 nm, λ = 280 nm). An LC-ESI-MS analysis of the reaction mixture (negative ion mode) provided MS profiles compatible with 4-CP dimerization and trimerization products. In particular, the peak at Rt = 29.3 min shows a molecular ion peak at *m*/*z* = 253.3, [M-H]^−^, whereas the peak at Rt = 21.8 min corresponds to a species with m/z = 379.2, [M-H]^−^ (Figures S4 and S5). The additional identification of isotopic peaks at [M-H+2n]^−^ (*n* = 1–3) both represent a strong indication of the presence in the molecules of multiple chlorine atoms and excluded that a dehalogenation reaction had taken place in these cases.

**Scheme 2:**
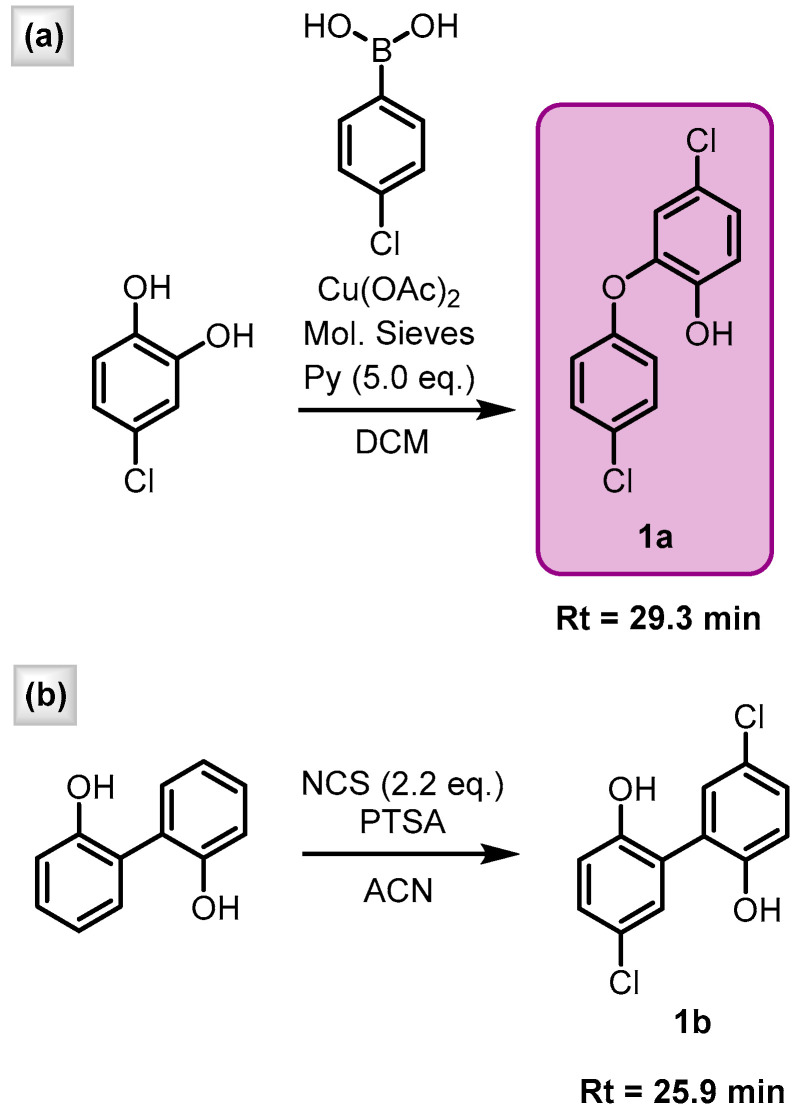
Synthesis and UHPLC Rt values of 4-chloro-2-(4-chlorophenoxy)phenol (**1a**) and 5,5’-dichloro-[1,1’-biphenyl]-2,2’-diol (**1b**).

To unequivocally identify whether dimeric and trimeric products, obtained by the Fe-MC6*a-catalyzed 4-CP oxidation, involved C-C or C-O covalent bonds, the corresponding 4-cholorophenol C-C and C-O dimers were synthesized by literature procedures (Scheme 2). 4-Chloro-2-(4-chlorophenoxy)phenol (**1a**, Scheme 2a) was obtained using the Evans method, relying on the copper(II)-promoted cross-coupling of 4-chlorophenylboronic acid and 4-chlorocatechol [55]. On the other hand, 5,5’-dichloro-[1,1’-biphenyl]-2,2’-diol (**1b**, Scheme 2b) was prepared using a slight variation of the synthetic method by Bentley et al. [56] (see Section 3 for the synthetic details). Figures S6–S8 report the NMR spectra of compounds **1a** and **1b**.

**Scheme 3:**
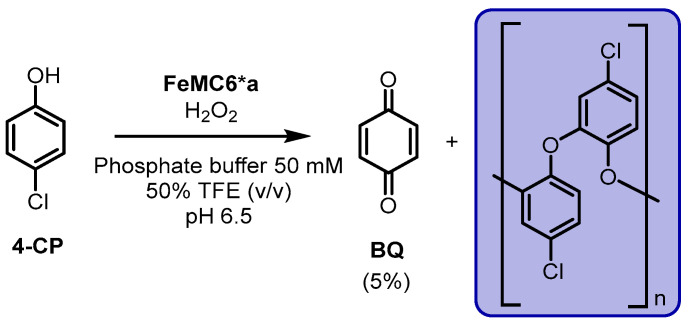
Reaction products in the Fe-MC6*a-catalyzed 4-CP oxidation.

The UHPLC analysis of compounds **1a** and **1b** (Scheme 2 and Figure S9) provided peaks at Rt values of 29.3 min and 25.9 min, respectively. This allowed to identify the peak at Rt = 29.3 min, derived from the Fe-MC6*a-catalyzed oxidation of 4-CP, as the dimeric 4-CP coupling product based on a C-O bond. Furthermore, the absence in the UHPLC reaction profile of the peak at Rt = 25.9 min, corresponding to the C-C dimer (Scheme 2, compound **1b**), indirectly suggests that the trimeric species was also based on C-O bonds (Scheme 3).

The formation of self-coupling products among 4-CP units recalls the peroxidase activity of horse heart myoglobin, which has been reported to catalyze the H_2_O_2_-dependent dimerization of 4-CP under conditions representative of oxidative stress [42]. In this case, the dimeric species was revealed to be based on a C-C bond in *ortho* positions to the OH groups. CCPO from *Caldariomyces fumago* and DHP from *Amphitrite ornata* were also described to provide a C-C-linked 4-CP dimer, along with variable amounts of BQ [45,57].

Having ascertained that the Fe-MC6*a-catalyzed 4-CP oxidation followed a different reaction route than 4-FP, an analysis of the reaction products was also carried out on 4-BP and 4-IP as substrates. As with 4-CP, with these substrates coupling products were observed, even though a lower conversion and a higher selectivity was found, as no traces of BQ were detected (Figures S10 and S11).

#### 2.2.3. Identification of the Insoluble Products in the Fe-MC6*a-Catalyzed 4-CP Oxidation

Having identified dimeric and trimeric coupling products occurring in the soluble fraction of the 4-CP oxidation reaction mixture, MALDI analysis (positive ion mode) then enabled us to suggest the nature of the insoluble material formed in the reaction. A family of oligomers, including up to fourteen 4-CP units and one terminal BQ moiety, was evidenced in the MALDI-MS spectrum (*m*/*z* 1914, [M + H]^+^; 1936, [M + Na]^+^; Figure S12).

**Figure 7:**
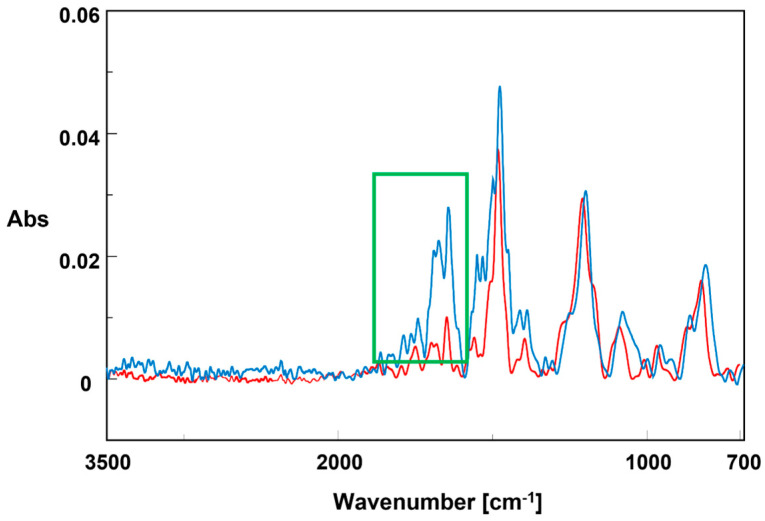
Comparison of FT-IR spectra referring to 4-CP polymerization resulting from Fe-MC6^*^a catalysis (red spectrum) or HRP catalysis (light-blue spectrum). The most relevant differences in the two spectra are highlighted in the green square.

These structures are in agreement with a previous hypothesis provided by other authors and referring to peroxidase-promoted halophenol polymerization [58]. A subsequent FT-IR analysis (ATR mode) of the insoluble products (Figure 7, red line) provided further support to the formation of oligomers based on C-O bonds. The presence in the spectrum of bands at ≈800 cm^−1^ is diagnostic of the occurrence of C-Cl bonds in the polymer structure. Furthermore, broad bands at approximately 3300 cm^−1^, typically referring to OH groups in phenols and in 4-CP, were missing. Finally, the minimal presence of bands at approximately 1700 cm^−1^ (compatible with CO bond stretching vibrations from benzoquinone moieties [59]) support the hypothesis of a “capping” reaction of the 4-CP oligomers, involving the dehalogenative oxidation of terminal 4-CP moieties to quinones.

It is well known that HRP is able to promote the H_2_O_2_-mediated polymerization of phenols and their derivatives, including 4-CP, providing insoluble products, based on a mixture of phenylene (C-C) and oxyphenylene (C-O) units [60,61,62]. This finding has been at the core of numerous research projects aimed at defining a protocol for the removal of 4-CP and other pollutants from wastewater [63,64,65]. To compare the regioselectivity of our artificial metalloenzyme with that of the HRP-catalyzed reaction, the conversion of 4-CP catalyzed by HRP was carried out, using optimal working conditions for the natural enzyme ([HRP] = 1.0 × 10^−3^ mM, [4CP] = 2.5 mM, [H_2_O_2_] = 2.5 mM, phosphate buffer 100 mM, and pH 6) [66]. An FT-IR analysis of the insoluble residue obtained in this reaction is reported in Figure 7 (light-blue line). Similar to the Fe-MC6^*^a-catalyzed reaction, bands at ≈800 cm^−1^, referring to C-Cl bond stretching vibrations, were detected. Bands at 3300 cm^−1^ (OH bond stretching) were also absent in this case. Differently from the product obtained by Fe-MC6*a catalysis, herein larger and more intense bands ranging from 1800 to 1600 cm^−1^ were detected (Figure 7, light-blue line).

Thus, a comparison of the FT-IR spectra of the insoluble products obtained from the two different catalysts highlighted several common features but also some remarkable differences. The observation that the IR spectrum of the Fe-MC6*a catalysis product was characterized by the absence of phenolic groups with a minimal presence of quinone moieties suggests that the artificial enzyme is able to promote selective transformations, leading to polymerization products having high structural homogeneity. Conversely, in the case of HRP, the presence of intense bands referring to C=O bond stretching (Figure 7, green square) along with those dealing with C-Cl bond stretching suggests that the polymerization products, characterized by a higher structural diversity, were formed, resulting from both self-coupling and dehalogenative oxidation reactions. Furthermore, the absence of bands referring to OH groups’ stretching vibrations suggests that side oxidation reactions of underivatized OH groups into quinones occurred.

### 2.3. Fe-MC6*a Chemodivergent Transformations in the Oxidation of Halophenols

The results presented above show that Fe-MC6*a catalysis exhibits peculiar features among both natural and artificial metalloenzymes. While peroxidases (HRP and CCPO) promote 4-halophenol oxidation leading to mixtures of both BQ and various radical-promoted coupling products (composed of both C-O and C-C bonds) independently from halogen’s nature, the globin-like peroxidase DHP has a limited effect on 4-FP and 4-CP. Further, myoglobin (Mb) is able to transform 4-CP into a mixture of BQ and the C-C-linked 4-CP dimer. Among simplified metalloenzymes, MP8 enables the conversion of all 4-halophenols into BQ. In this scenario, Fe-MC6*a demonstrated the ability to promote substrate-dependent chemodivergent transformations, selectively providing BQ from 4-FP and coupling products from 4-CP. Additionally, in the latter case, catalysis provided oligomerization products based on C-O rather than C-C bonds, which were provided with unprecedented regioselectivity. The detection of oligomerization products is an indication that the reaction proceeds through the formation of radical species, as previously suggested by Dawson and coworkers [45]. In an effort to detect the 4-chlorophenoxyl radical, 4-CP oxidation was performed in the presence of the radical scavenger DMPO (5,5-dimethyl-1-pyrroline-*N*-oxide) [67] and monitored by GC-MS analysis (Figures S13 and S14). While in the FeMC6*a-catalyzed 4-CP oxidation a significant substrate consumption was detected (Figure S13), the same reaction was totally inhibited when carried out in the presence of a large excess of DMPO (50 eq.; Figure S14).

**Scheme 4:**
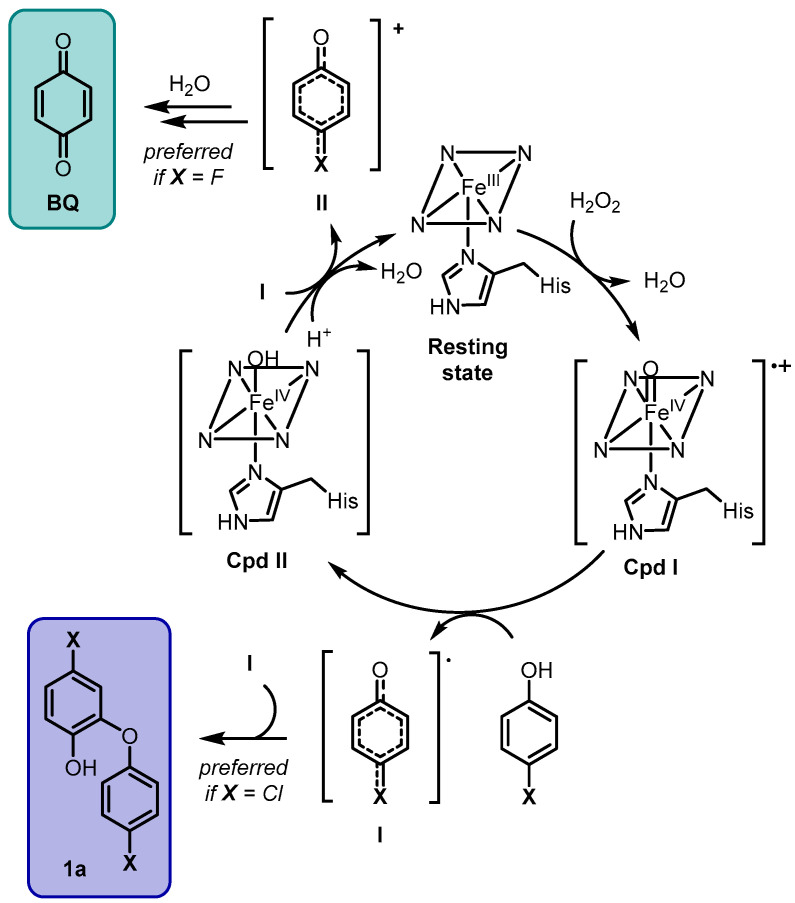
Fe-MC6*a chemodivergent catalysis with 4-halophenols.

Based on the above analysis of the product distribution obtained during the Fe-MC6*a-catalyzed oxidation of 4-halophenols, some preliminary mechanistic considerations may be proposed. First, the formation of oligomeric products is consistent with the involvement of radicals formed during the reaction. Second, the chemodivergent outcome of the reactions suggests that different substrates must have followed different oxidation routes. According to known mechanistic studies on natural metalloenzymes [44,45], as well as on the latest findings on the Fe-MC6*a-catalyzed oxidation of halophenols [38], the most plausible reaction mechanism for the Fe-MC6*a-catalyzed 4-halophenol oxidation (Scheme 4) involves the initial formation of Cpd I, characterized by the presence of an oxoferryl radical cation [(Fe(IV)=O)por^+^]. This intermediate species is obtained upon H_2_O_2_ activation of the catalyst in the resting state. Cpd I formation of Fe-MC6*a has already been demonstrated previously by spectroscopic studies [36,38]. Cpd I is thought to promote a one-electron oxidation, providing the phenoxy radical species **I** and Compound II (Cpd II), corresponding to the ferryl heme lacking the porphyrin radical, whose formation was postulated in previous studies [38]. Self-coupling reactions among radicals **I** are expected to provide dimer **1a** and the corresponding oligomers. On the other hand, a further one-electron oxidation from **I** yields a positively charged dienone **II**, which is converted into BQ upon reaction with a water molecule (Scheme 4).

Based on this catalytic cycle, we reasoned that the conversion of radical **I** into cation **II** represents the key step affecting the chemodivergent outcome of the reaction. Indeed, the accumulation of radical **I** is expected to favor the formation of self-coupling products, while the access to a higher concentration of **II** would enable the selective conversion of the 4-halophenol into BQ (Scheme 4). A suitable enzyme-driven accumulation of one of the two chemical species can be hypothesized to have a role in the product distribution of this reaction. Considering the uncatalyzed reaction, the formation of cation **II** is thermodynamically favored only in the reaction of 4-FP, owing to the more effective fluorine-mediated delocalization of the positive charge by electron-donating resonance effect. Conversely, no preference for radical **I** or cation **II** is expected when 4-CP, 4-BP and 4-IP are used as substrates. In the enzyme-catalyzed reaction, the preferential accumulation of single intermediates may occur, thus overcoming the stereoelectronic features of each substrate. This would imply the enzyme’s ability to stabilize one chemical species over another one. More mechanistic studies will be performed to shed light on the molecular basis for the different pathways observed, aiming at establishing the role of our artificial catalyst over all existing metalloenzymes used for the same catalytic purposes.

## 3. Materials and Methods

All solvents were supplied by Romil (Cambridge, UK). All reagents including halophenols, MPS, 2,2’-biphenol, *p*-toluenesulfonic acid (PTSA), *N*-chlorosuccinimide (NCS), 4-chlorocatechol, 4-chlorophenylboronic acid, hydrogen peroxide (H_2_O_2_) solution (30% (*v*/*v*)), α-cyano-4-hydroxycinnamic acid (α-CHCA) and phosphate salts (mono- and dibasic) were purchased from Merck (Merck KGaA, Darmstadt, Germany). Fe(III)-MC6*a was synthesized, purified and characterized as previously described [36].

UV–Vis spectra were recorded on a Cary Varian 60 Probe UV spectrophotometer equipped with a temperature controller (Varian, Palo Alto, CA, USA). All measurements were performed at 25 °C in 50 mM phosphate buffer (pH 6.5) with 50% TFE (*v*/*v*), unless otherwise specified. Quartz cuvettes with a path length of 1.0 cm were used for the measurements. The wavelength scans were performed from 200 to 800 nm, with a 600 nm min^−1^ scan speed. All data were blank subtracted. The Fe(III)-MC6*a concentration was determined by UV–Vis spectroscopy using ε = 1.17 × 10^5^ M^−1^ cm^−1^ in H_2_O 0.1% TFA (*v*/*v*) (λ = 387 nm). The data analysis was performed using Origin Pro, version 9.0 (Origin Lab Corporation, Northampton, MA, USA).

HPLC analyses were performed with a Shimadzu LC-20ADxr equipped with an SPDM20A diode-array detector (Shimadzu Corporation, Kyoto, Japan). ESI-MS spectra were recorded on a Shimadzu LCMS-8040 system with ESI interface, triple-quadrupole mass analyzer (Shimadzu Corporation, Kyoto, Japan) and Shimadzu LC-MS solution Workstation (version 5.97) software for data processing. All analyses were performed with a Kinetex Phenyl-Hexyl column (150 mm × 4.6 mm, 5 μm) using water with 0.1% TFA (A) and acetonitrile with 0.1% TFA (B) as the eluents. A linear gradient from 5 to 95% B over 35 min at a flow rate of 1.85 mL/min was used as the elution method.

GC-MS analysis was performed on a Shimadzu GCMS-QP2010 SE system equipped with an EI-MS source, a quadrupole array as an MS analyzer (Shimadzu Corporation, Kyoto, Japan) and a Shimadzu GC-MS solution Workstation (version 4.20) for data processing. A Restek Rxi-5Sil-MS column was used, with helium being selected as the carrier gas. A linear gradient from 50 °C to 300 °C with a rate of 24 °C·min^−1^ was used. The mass spectra were recorded in an *m*/*z* range between 50 and 450.

Matrix-assisted laser desorption/ionization (MALDI)-MS experiments were performed on a 5800 MALDI time-of-flight (TOF)/TOF AB SCIEX equipped with a nitrogen laser (337 nm) (SCIEX, Framingham, MA, USA). The laser power was set to 3500 V for MS spectra acquisition. Each spectrum represents the sum of 10,000 laser pulses from randomly chosen spots per sample position. The data are reported as monoisotopic masses.

NMR spectra were acquired using Bruker Avance 600 or 400 MHz spectrometers (Karlsruhe, Germany). All NMR experiments were carried out at 298 K. All spectra were processed with MestreNova (version 14.2.2).

FT-IR-ATR spectra were recorded on solid phase by a JASCO FT/IR−430 spectrophotometer (JASCO Europe, Lecco, Italy) in the 4000–700 cm^−1^ range, using HATR crystal zinc selenide (Flat Plate, PN 022–2020–45) by PIKE Technologies. The resolution was 2 cm^−1^. Each spectrum was recorded 128 times.

### 3.1. Fe-MC6*a-Catalyzed Oxidations of Halophenols: General Procedure

For UV–Vis analysis: a solution of H_2_O_2_ (0.25 mM) was added in one portion at room temperature to a stirring solution of the halophenol (0.25 mM) and Fe(III)-MC6*a (6.35 × 10^−4^ mM) in 50 mM sodium phosphate (pH 6.5) with TFE (1:1 *v*/*v*). The reaction was carried out in an overall reaction volume of 0.66 mL. The reaction monitoring was performed by acquiring UV–Vis spectra over 100 min (wavelength window: 200–800 nm; scan speed: 600 nm/min).

For chromatographic analysis: a solution of H_2_O_2_ (2.5 or 3.75 mM) was added in one portion at room temperature to a stirring solution of the halophenol (2.5 or 3.75 mM), MPS (2.5 or 3.75 mM) and Fe(III)-MC6*a (6.25 × 10^−3^ mM or 6.25 × 10^−5^ mM) in 50 mM sodium phosphate (pH 6.5) with TFE (1:1 *v*/*v*). The reaction was carried out in an overall reaction volume of 1 mL. For GC-MS analysis, 50 μL aliquots were withdrawn before (t = 0) and after (t = 10, 20 and 30 min) H_2_O_2_ addition and transferred to 2.0 mL Eppendorf tubes. The reaction mixtures were quenched by the addition of a H_2_O 0.1% TFA (*v*/*v*) (50 μL) and extracted with dichloromethane (100 μL). Organic layers were dried (Na_2_SO_4_), centrifuged at 4000 rpm and the supernatants were injected. For HPLC analysis, 50 μL aliquots were withdrawn before (t = 0) and after (t = 10, 20 and 30 min) H_2_O_2_ addition and transferred to 1.5 mL Eppendorf tubes. The reaction mixtures were quenched by the addition of a H_2_O 0.1% TFA (*v*/*v*) (50 μL) and injected.

For NMR analysis: a solution of H_2_O_2_ (2.5 mM) was added in one portion at room temperature to a solution of 4-CP (2.5 mM), MPS (2.5 mM) and Fe(III)-MC6*a (6.25 × 10^−3^ mM) in 50 mM sodium phosphate (pH 6.5), TFE and D_2_O (40:50:10 *v*/*v*/v). The reaction monitoring was performed by acquiring ^1^H NMR spectra over time. A confirmation of the identity of BQ was obtained by comparison with the ^1^H NMR spectrum of a commercial sample, acquired under the same conditions.

### 3.2. TON Determination

The turnover number was determined by GC-MS analysis of the reaction, measuring the conversion degree based on the maximal substrate consumption. Following the procedure reported in the previous paragraph, total ion current (TIC) chromatograms were acquired. The peak areas corresponding to the halophenol and MPS were integrated for all reaction times. The conversion degree was calculated using the following equation:

$$\mathrm{Conversion}\;\left(\%\right)=\frac{{\left(\frac{A_{\mathrm{sub}}}{A_{I.\mathrm{Std}.}}\right)}_{0}-{\left(\frac{A_{\mathrm{sub}}}{A_{I.\mathrm{Std}.}}\right)}_{x}}{{\left(\frac{A_{\mathrm{sub}}}{A_{I.\mathrm{Std}.}}\right)}_{0}}\;\cdot 100$$

where A_sub_ and A_I.Std._ are the peak areas of the substrate and the internal standard, respectively, in the GC-MS TIC chromatogram. The subscript 0 indicates the trace acquired prior to H_2_O_2_ addition, while the subscript *x* is a specific time during the reaction. Based on the conversion degree, the TON value was calculated as the ratio between the number of converted substrate molecules and the number of catalyst molecules. The mean TON values along with the corresponding standard deviations are reported in Table 1.

### 3.3. Synthesis of Compounds 1a and 1b

4-Chloro-2-(4-chlorophenoxy)phenol (1a). Compound **1a** was synthesized from 4-chlorocatechol and 4-chlorophenylboronic acid according to the procedure of Suzuki et al. [55,68]. Spectral data of compound **1a** are fully in line with those reported in the literature [68]. ^1^H NMR (CDCl_3_, 600 MHz), δ: 5.60 (bs, 1H), 6.78 (d, J = 3.8 Hz, 1H), 6.83 (dd, J = 2.3, 8.6 Hz, 1H), 6.95 (d, J = 8.8 Hz, 2H), 7.06 (d, J = 2.4 Hz, 1H), and 7.31 (d, J = 8.7 Hz, 2H).

5,5′-Dichloro-[1,1′-biphenyl]-2,2′-diol (1b). A solution of 2,2′-biphenol (200 mg, 1.07 mmol), PTSA (450 mg, 2.36 mmol) and NCS (313 mg, 2.36 mmol) in 10 mL of ACN was stirred at room temperature for 16 h. The reaction was quenched with water and the mixture extracted with dichloromethane. The combined organic layers were dried over Na_2_SO_4_ and concentrated under vacuum. Purification by flash chromatography on silica gel (hexane/ethyl acetate = 9/1) afforded 190 mg (0.43 mmol, 40%) of a white solid. ^1^H NMR (ACN-*d*_3_, 600 MHz), δ: 6.93 (d, J = 8.5 Hz, 2H), 7.15 (bs, 2H), 7.24 (d, J = 2.2 Hz, 2H), and 7.26 (dd, J = 2.5, 8.5 Hz, 2H). ^13^C NMR (ACN-*d*_3_, 100 MHz): 118.6, 125.3, 126.9, 130.0, 131.8, and 153.8 ppm.

### 3.4. Analysis of Polymerization Products in the Enzyme-Catalyzed 4-CP Oxidation

The polymerization reactions were performed separately using Fe-MC6*a and HRP as catalysts. Compared to previous conditions (Figure 6) [66], higher concentrations of biocatalysts were herein used to enable the full conversion of the starting material: (a) [Fe-MC6*a] = 1.0 × 10^−2^ mM; [4-CP] = 2.5 mM; [H_2_O_2_] = 2.5 mM in 50 mM phosphate buffer (pH 6.5) with 50% TFE (*v*/*v*) at 25 °C; (b) [HRP] = 1.0 × 10^−3^ mM; [4-CP] = 2.5 mM; [H_2_O_2_] = 2.5 mM in 100 mM phosphate buffer (pH 6) at 30 °C. In both cases, the reactions were carried out in overall volumes of 10 mL. After 1 h, control HPLC runs indicated that no residual 4-CP was present in both reaction mixtures.

Afterwards, reaction (a) was concentrated under reduced pressure to remove TFE. The resulting suspension was centrifuged; the precipitate was washed several times with water and then lyophilized to obtain a black powder. Analogously, the reaction mixture (b) was centrifuged; the precipitate was washed several times with water and then lyophilized, obtaining a black powder.

MALDI-MS analysis: a suspension of polymerization products obtained by Fe-MC6*a or HRP catalysis (0.5 μL) in 7:3 ACN:H_2_O (*v*/*v*) was mixed with a 20 mg/mL solution of α-CHCA in 7:3 ACN:H_2_O (*v*/*v*) (0.5 μL) and deposited onto a metal target plate for MALDI-MS analysis. The MS spectra were acquired in the positive reflectron mode using a mass (*m*/*z*) range of 200–4000 Da.

FT-IR analysis: a few milligrams of the black powders were placed onto the ATR cell of the FT-IR spectrometer and analyzed.

## 4. Conclusions

This work represents a further extension of our research aimed at evaluating the catalytic potential of the miniaturized metalloenzyme Fe-MC6*a. The catalytic behavior of Fe-MC6*a toward the oxidation of differently halogenated phenols was herein screened. The complete characterization of the reaction products for the 4-halophenols case study highlighted interesting catalyst performances. Fe-MC6*a catalyzed the selective oxidative dehalogenation of 4-FP into BQ, while the same reaction with 4-CP, 4-BP and 4-IP resulted in the formation of higher molecular weight compounds. After UHPLC, UV–Vis, NMR, MS and IR analyses, we collected strong indications that oligomers based on C-O bonds were selectively formed during 4-CP oxidation. Differently from all natural and artificial metalloenzymes tested so far in the same reaction, Fe-MC6*a thus demonstrated a distinctive enzymatic character, promoting substrate-dependent chemodivergent transformations with a very high selectivity. This peculiarity is well suited for synthetic applications toward complex halophenol-derived synthons. Even though the preferred formation of specific intermediates is supposed to be at the origin of this catalytic behavior, in-depth mechanistic studies, currently under course, are needed to clarify the peculiar role of this catalyst in respect to the natural and artificial metalloenzymes known so far.

## Figures and Tables

**Table 1:** TON values of the Fe-MC6*a-catalyzed oxidation of halophenols.

Entry	Substrate	Abbreviation	TON
**1**	2-Chlorophenol	2-CP	310 ± 19
**2**	4-Fluorophenol ^1^	4-FP	503 ± 10
**3**	4-Chlorophenol	4-CP	325 ± 22
**4**	4-Bromophenol	4-BP	235 ± 10
**5**	4-Iodophenol	4-IP	208 ± 30
**6**	2,4-Dichlorophenol ^2^	2,4-DCP	2588 ± 103
**7**	2,4,6-Trifluorophenol ^2^	2,4,6-TFP	3825 ± 171
**8**	2,4,6-Trichlorophenol ^2^	2,4,6-TCP	3850 ± 148
**9**	Pentafluorophenol	PFP	142 ± 27
**10**	Pentachlorophenol	PCP	186 ± 64

General conditions: [Fe-MC6*a] = 6.25 × 10^−3^ mM; [substrate] = 2.5 mM; [H_2_O_2_] = 2.5 mM; 50 mM phosphate buffer, pH 6.5 with 50% (*v*/*v*) TFE. MPS was used as an internal standard ([MPS] = 2.5 mM). ^1^ In this case, [4-FP] = [H_2_O_2_] = 3.75 mM; the remaining parameters were left unchanged. ^2^ In this case, [Fe-MC6*a] = 6.25 × 10^−5^ mM, while the remaining parameters were left unchanged.

## Data Availability

All relevant data supporting the findings of this study are included in the article and/or in supplementary information files. Data are also available upon request from the corresponding author.

## Supplementary Materials

The following supporting information can be downloaded at: https://www.mdpi.com/article/10.3390/ijms24098058/s1.
