# Supplementary material for: Selective Oxidation of Halophenols Catalyzed by an Artificial Miniaturized Peroxidase
Source: Int J Mol Sci. 2023 Apr 29;24(9):8058. doi: 10.3390/ijms24098058 (PMC10178546; doi:10.3390/ijms24098058)
Supplement: Supplementary file 1 [file ijms-24-08058-s001.zip › ijms-2338925-supplementary.pdf]

SUPPORTING INFORMATION

# Selective oxidation of halophenols catalyzed by an artificial miniaturized peroxidase

*Daniele D'Alonzo, Maria De Fenza, Vincenzo Pavone, Angela Lombardi and Flavia Nastri**

Department of Chemical Sciences, University of Napoli Federico II, Via Cintia, 80126 Napoli, Italy

flavia.nastri@unina.it

## Table of Contents

**Figure S1.** EI-MS analysis of the reaction product of the Fe-MC6*a-catalyzed 4-FP oxidation.

**Figure S2.** Fe-MC6*a-catalyzed 4-CP oxidation: $^{1}$H NMR spectrum of the reaction mixture.

**Figure S3**. UV-Vis spectral profiles of 4-CP and the corresponding dimeric and trimeric species.

**Figure S4**. Fe-MC6*a-catalyzed 4-CP oxidation: ESI-MS spectrum of the dimeric compound.

**Figure S5**. Fe-MC6*a-catalyzed 4-CP oxidation: ESI-MS spectrum of the trimeric compound.

**Figure S6**. $^{1}$H NMR spectrum of compound **1a**.

**Figure S7**. $^{1}$H NMR spectrum of compound **1b**.

**Figure S8**. $^{13}$C NMR spectrum of compound **1b**.

**Figure S9**. UHPLC profiles of compounds **1a** and **1b**.

**Figure S10**. Fe-MC6*a-catalyzed 4-BP oxidation: UHPLC profile of the reaction mixture.

**Figure S11**. Fe-MC6*a-catalyzed 4-IP oxidation: UHPLC profile of the reaction mixture.

**Figure S12**. Fe-MC6*a-catalyzed 4-CP oxidation: MALDI spectrum of the insoluble products.

**Figure S13**. Fe-MC6*a-catalyzed 4-CP oxidation: GC analysis.

**Figure S14**. Fe-MC6*a-catalyzed 4-CP oxidation in the presence of DMPO: GC analysis.

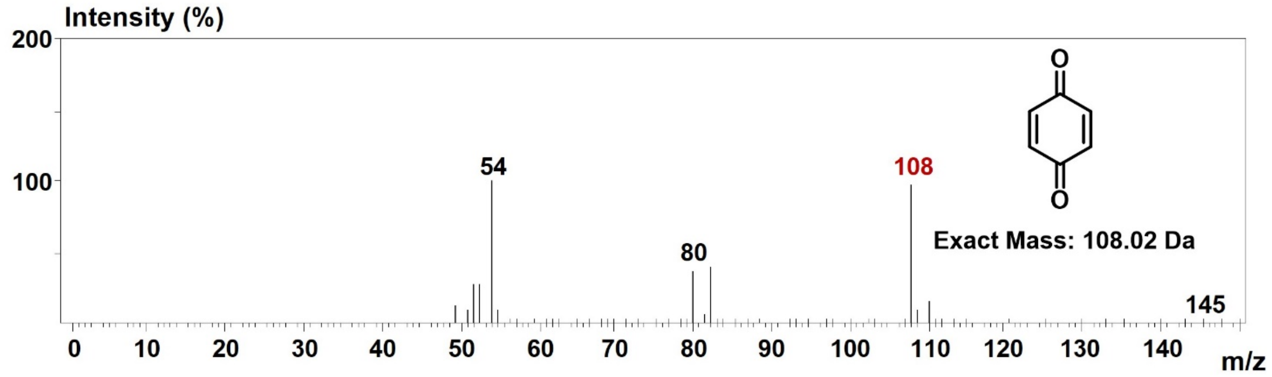


**Figure S1**. EI-MS analysis of the product obtained by the Fe-MC6*a-catalyzed 4FP oxidation in the presence of $H_2O_2$.

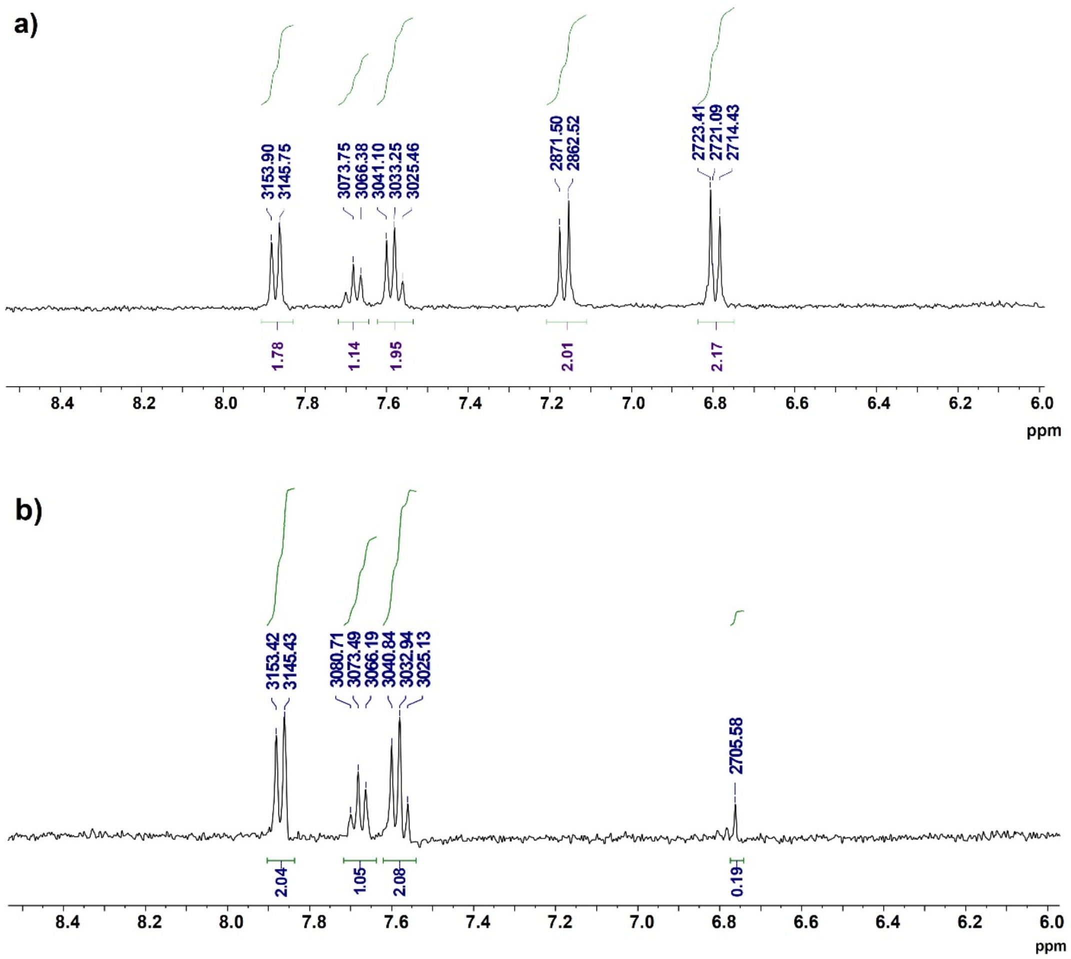


**Figure S2**. Close up views of the $^1H$ NMR spectra referred to the Fe-MC6*a-catalyzed 4-CP oxidation. Spectra were acquired at (a) t = 0 min and (b) t = 30 min from $H_2O_2$ addition. Reaction solvent was composed of phosphate buffer (50 mM), TFE and $D_2O$ (40:50:10 v/v/v).

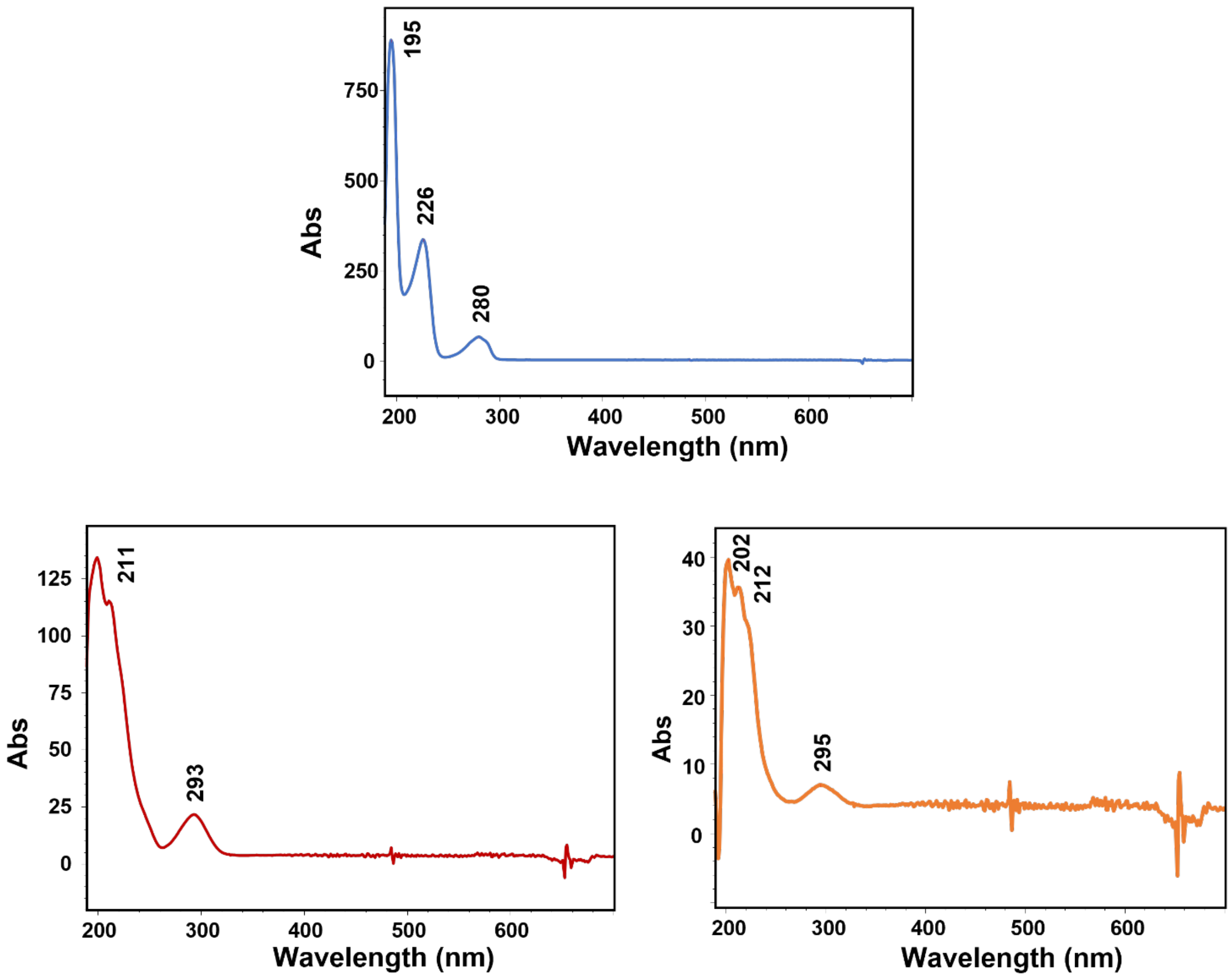


**Figure S3**. UV-Vis spectral profiles of the following products running in the UHPLC chromatogram (Figure 6): 4-CP (light blue curve, peak at Rt = 14.0 min), trimeric compound (dark red curve, peak at Rt = 21.8 min) and dimeric compound (orange curve, peak at Rt = 29.3 min).

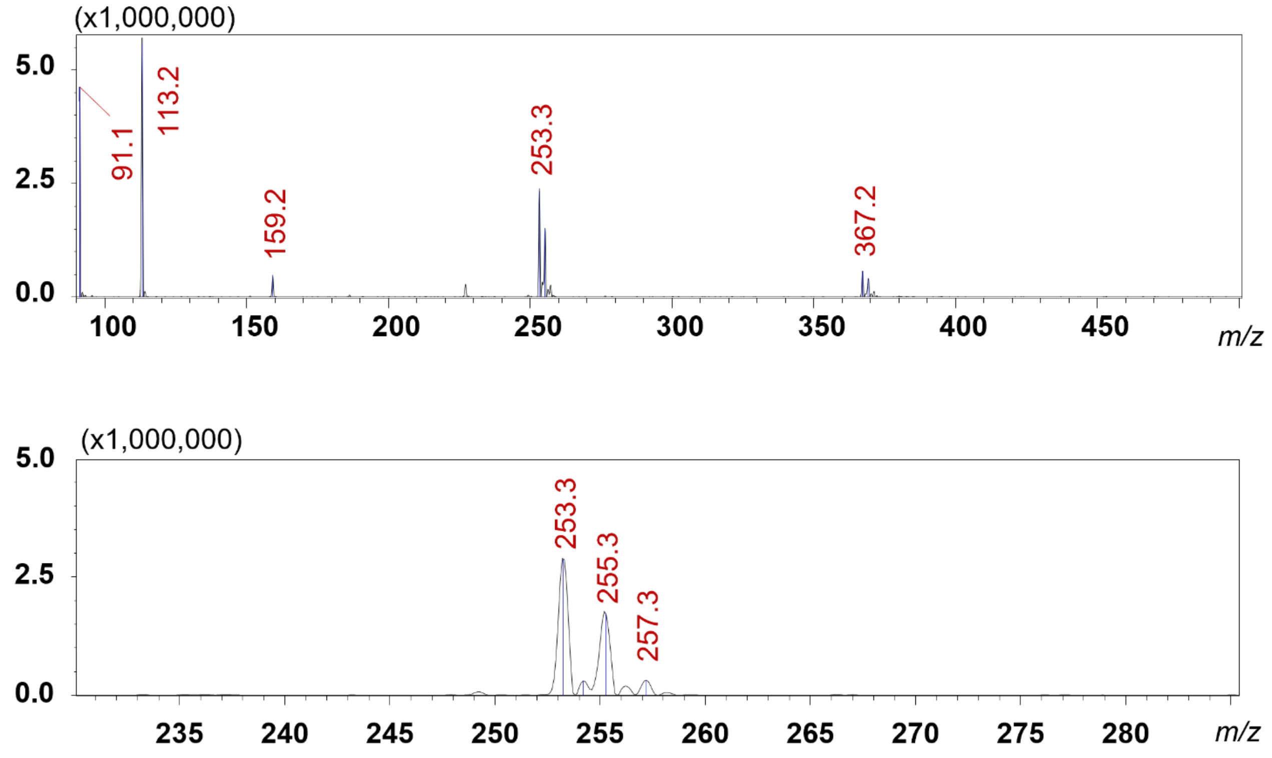


**Figure S4**. ESI-MS spectrum (negative ion mode, extracted from LC-MS analysis, peak at Rt = 29.3 min) of the dimeric compound obtained during 4-CP oxidation. The peak at m/z = 253.3 is referred to the molecular ion ($[M-H]^-$). In addition, peaks at m/z = 255 ($[M-H+2]^-$) and at m/z = 257.3 ($[M-H+4]^-$) are indicative of the presence of two chlorine atoms.

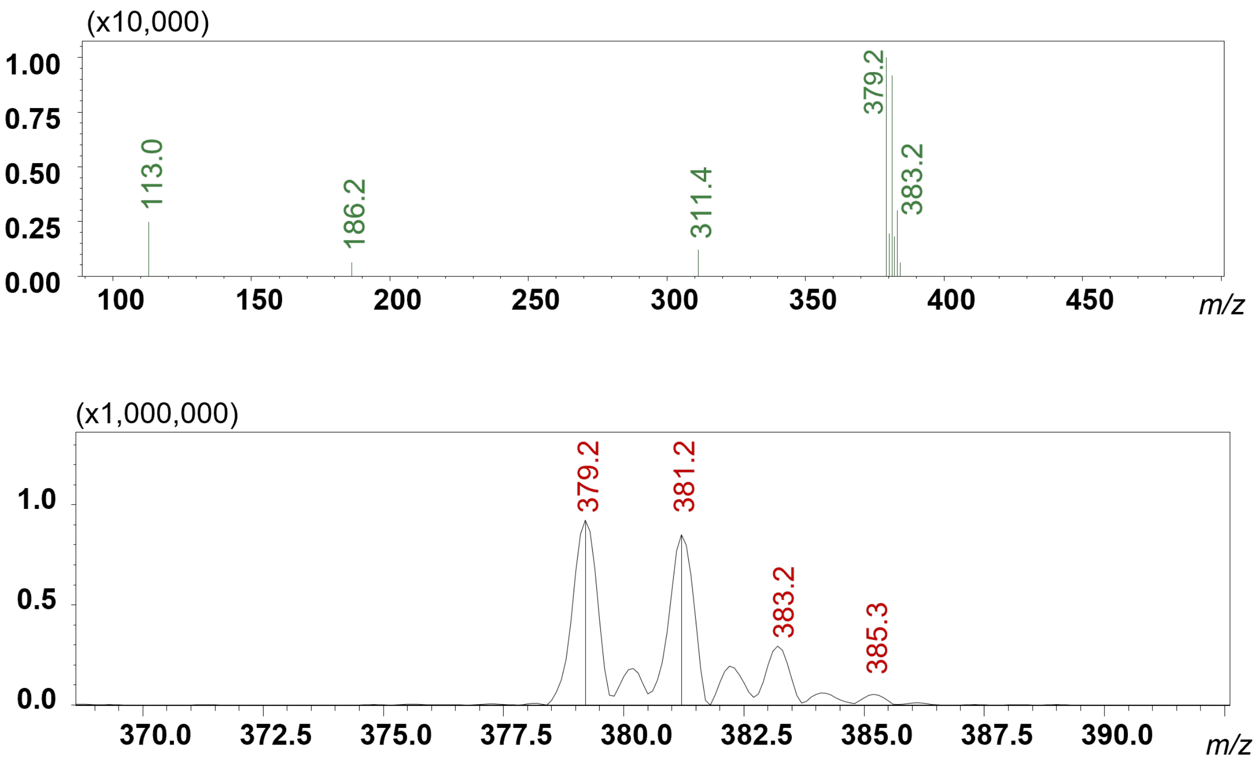


**Figure S5**. ESI-MS spectrum (negative ion mode, extracted from LC-MS analysis, peak at Rt = 21.8 min) of the trimeric compound obtained during 4-CP oxidation. The peak at m/z = 379.2 is referred to the molecular ion ($[M - H]^-$). In addition, peaks at m/z = 381.2 ($[M\text{-}H\text{+}2]^-$), m/z = 383.2 ($[M\text{-}H\text{+}4]^-$) and m/z = 385.3 ($[M\text{-}H\text{+}6]^-$) indicate the presence of three chlorine atoms.

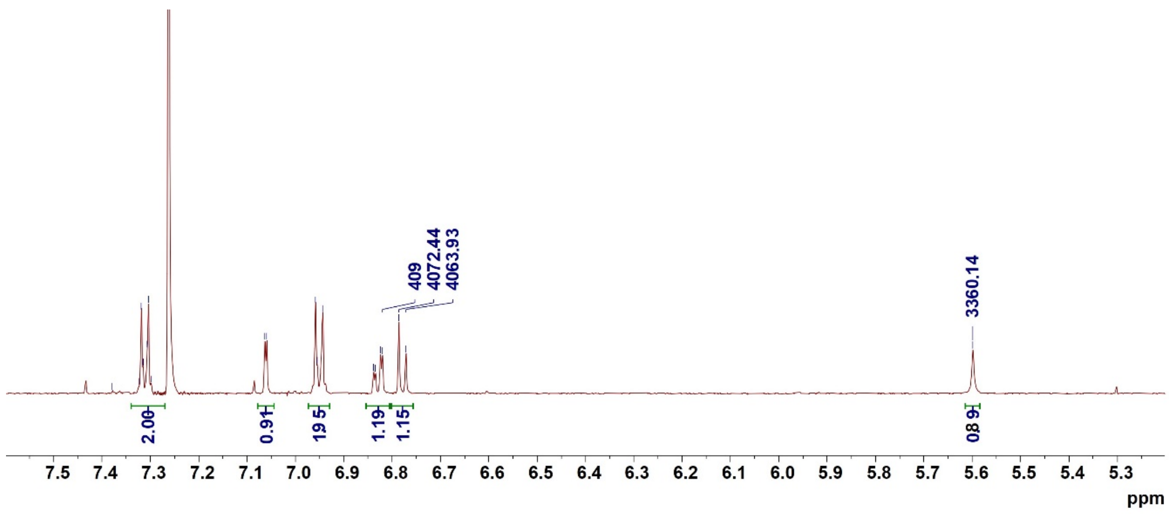


**Figure S6**. Close up view of the $^{1}$H NMR spectrum of compound **1a** ($CDCl_3$, 600 MHz).

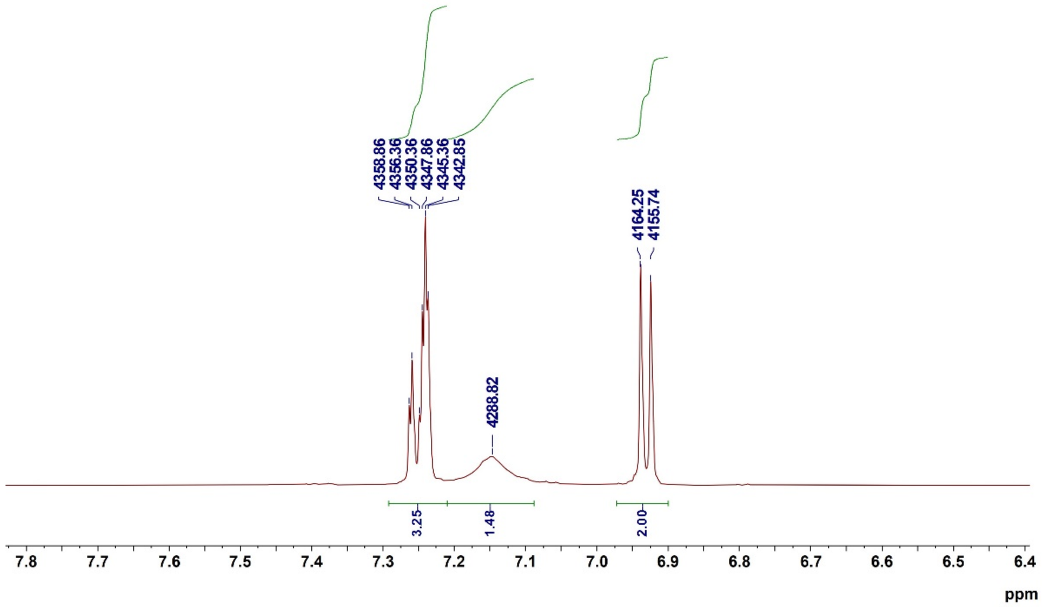


**Figure S7**. Close up view of the $^{1}$H NMR spectrum of compound **1b** (ACN-$d_3$, 600 MHz).

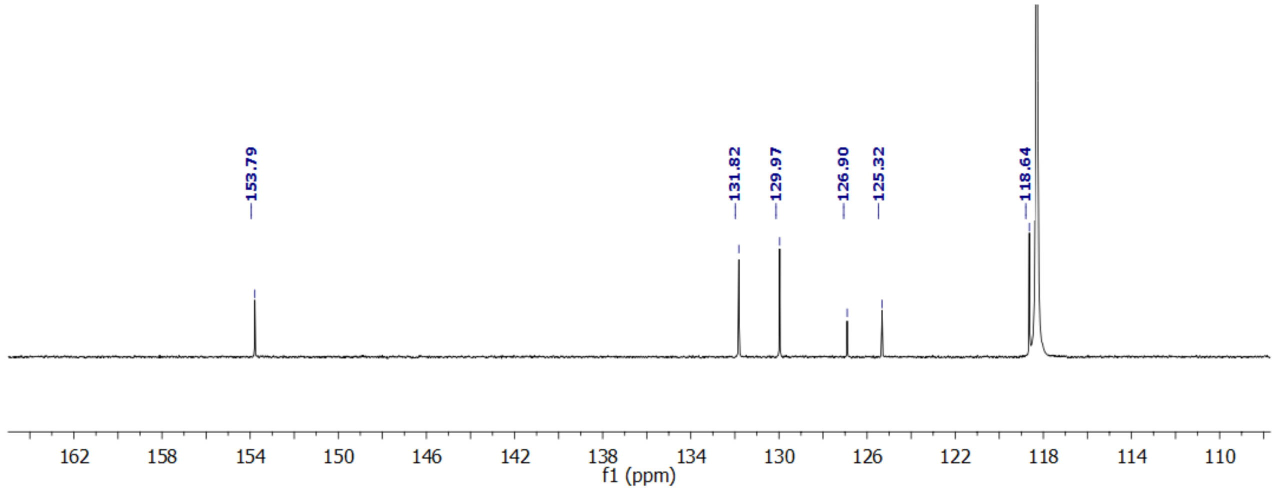


**Figure S8**. $^{13}$C NMR spectrum of compound **1b** (ACN-$d_3$, 100 MHz).

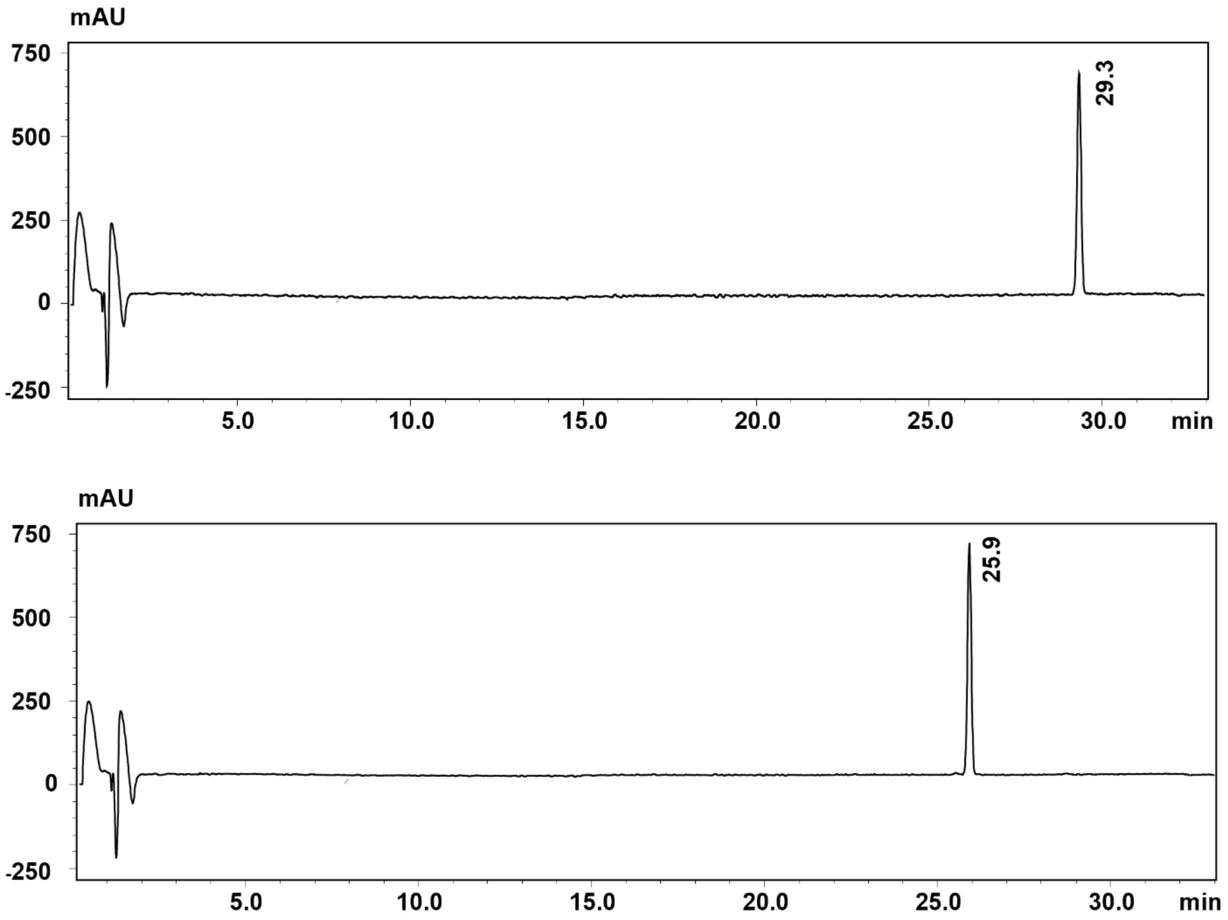


**Figure S9**. UHPLC runs of compounds **1a** (upper trace) and **1b** (lower trace).

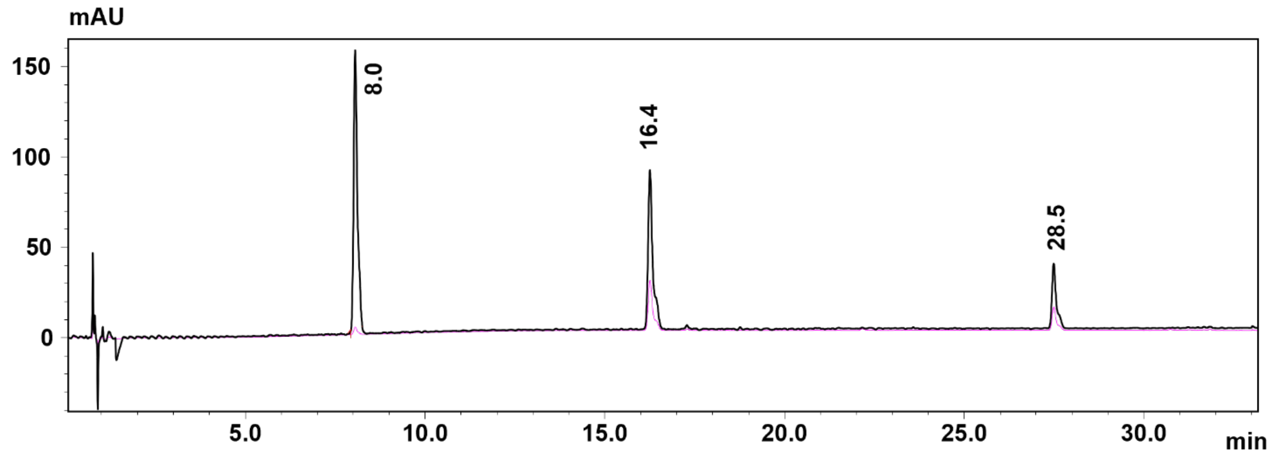


**Figure S10**. UHPLC traces (black line: λ = 220 nm, pink line: λ = 240 nm) of the Fe-MC6*a-catalyzed oxidation of 4-BP 30 min after $H_2O_2$ addition. The peak with Rt = 16.4 min corresponds to 4-BP; the peak with Rt = 8.0 min is MPS, used as internal standard. Reaction conditions: [Fe-MC6*a] = 6.25 x $10^{-6}$ M; [4-BP] = 2.5 mM; [$H_2O_2$] = 2.5 mM; phosphate buffer (50 mM) at pH 6.5 with 50% TFE (v/v).

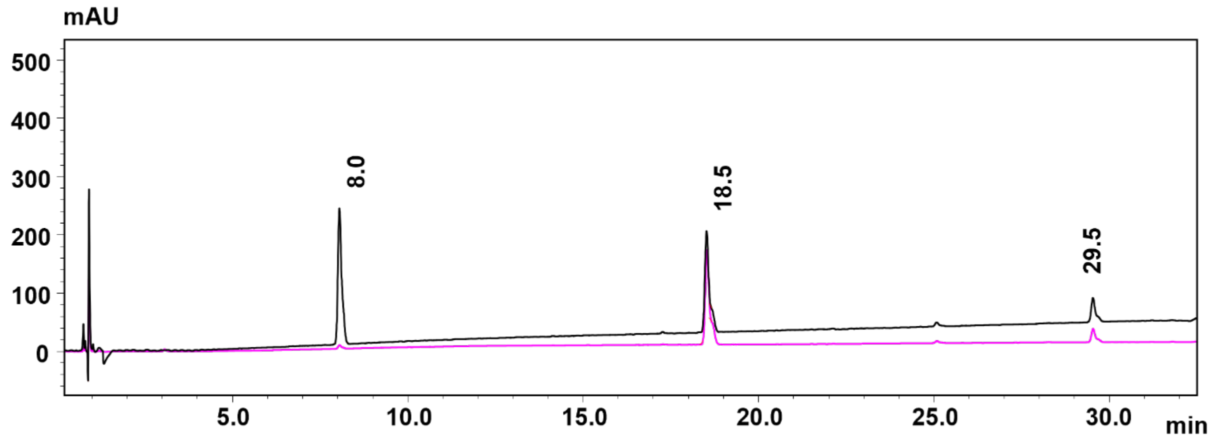


**Figure S11**. UHPLC traces (black line: λ = 220 nm, pink line: λ = 240 nm) of the Fe-MC6*a-catalyzed oxidation of 4-IP 30 min after $H_2O_2$ addition. The peak with Rt = 18.5 min corresponds to 4-iodophenol; the peak with Rt = 8.0 min is MPS, used as internal standard. The peak with Rt = 29.5 min corresponds to 4-IP dimer, as determined by LC-MS analysis. Reaction conditions: [Fe-MC6*a] = 6.25 x $10^{-6}$ M; [4-IP] = 2.5 mM; [$H_2O_2$] = 2.5 mM; phosphate buffer (50 mM) at pH 6.5 with 50% TFE (v/v).

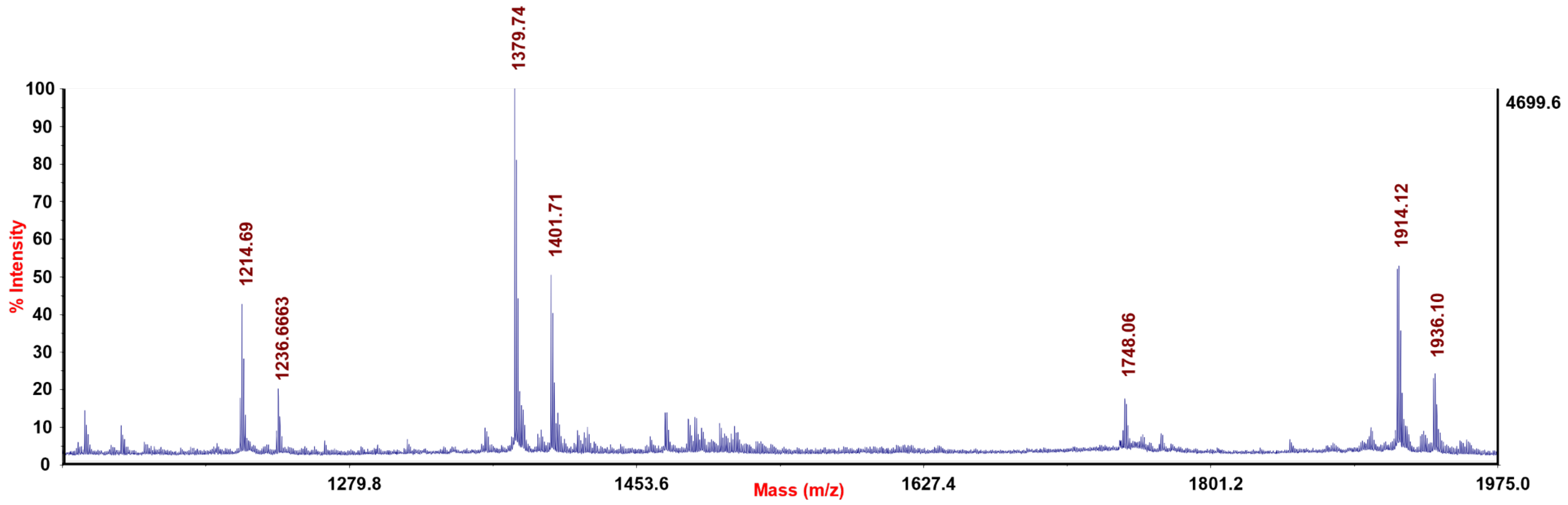


**Figure S12**. Close up view of the MALDI-MS spectrum of the insoluble material formed during the course of the Fe-MC6*a-catalyzed 4-CP oxidation.

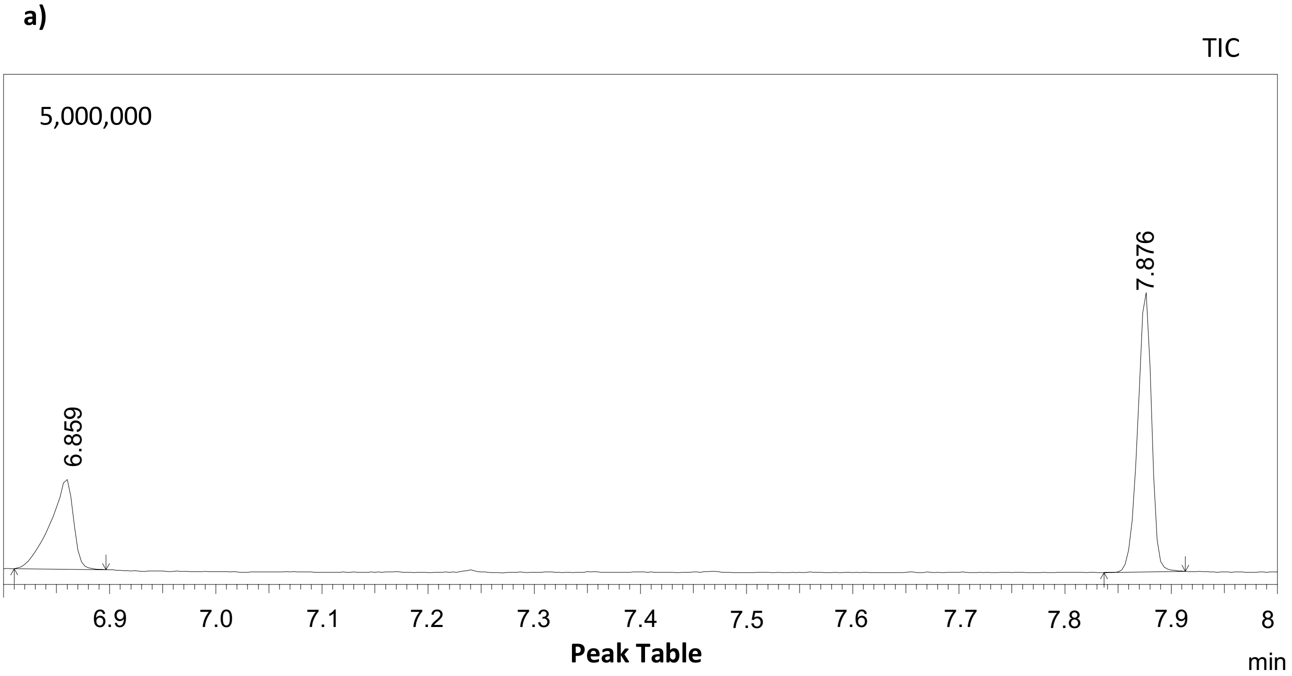


**Peak Table**

| Peak # | Compound | Rt (min) | Area |
|---|---|---|---|
| 1 | 4-CP | 6.859 | 1382473 |
| 2 | MPS | 7.876 | 2525874 |

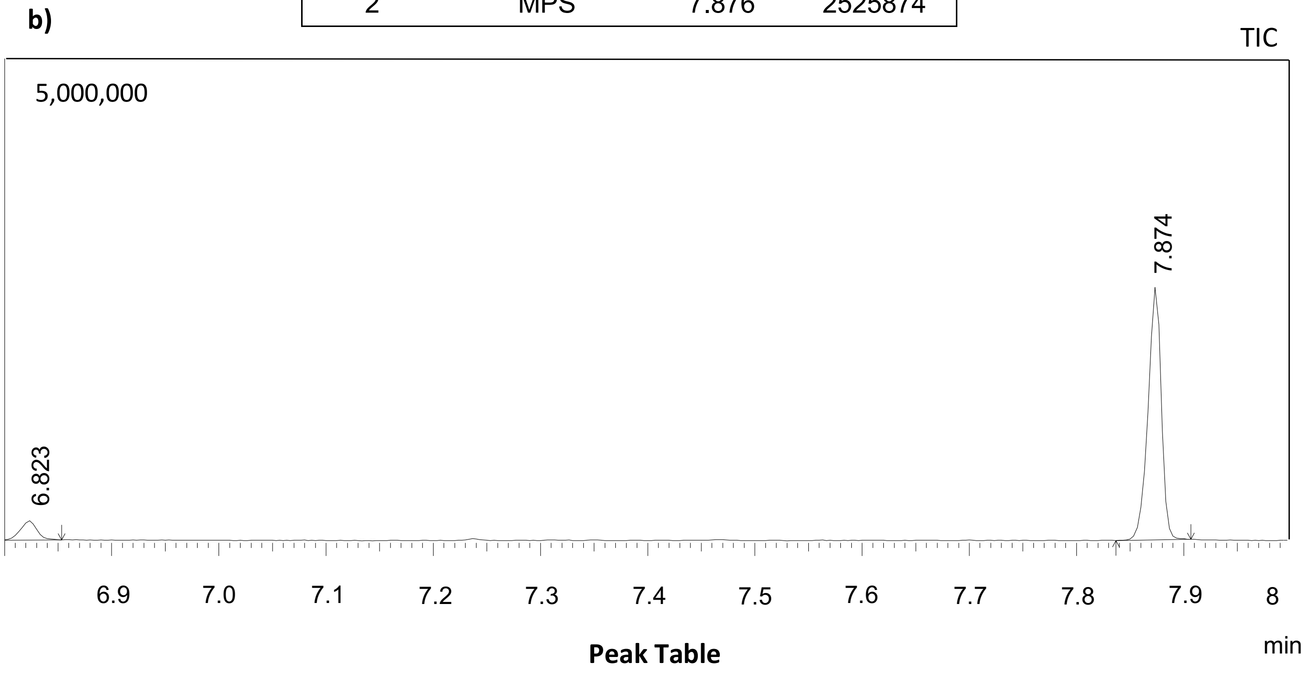


**Peak Table**

| Peak # | Compound | Rt (min) | Area |
|---|---|---|---|
| 1 | 4-CP | 6.823 | 213410 |
| 2 | MPS | 7.874 | 2249164 |

**Figure S13**. GC-MS analysis of the FeMC6*a-catalyzed 4-CP oxidation (a) before and (b) 30 min after $H_2O_2$ addition. *Reaction conditions*: [Fe-MC6*a] = 1.0 x $10^{-3}$ mM; [4-CP] = 2.5 mM; [$H_2O_2$] = 2.5 mM; [MPS] = 2.5 mM; phosphate buffer (50 mM) at pH 6.5 with 50% TFE (v/v).

**a)**

TIC

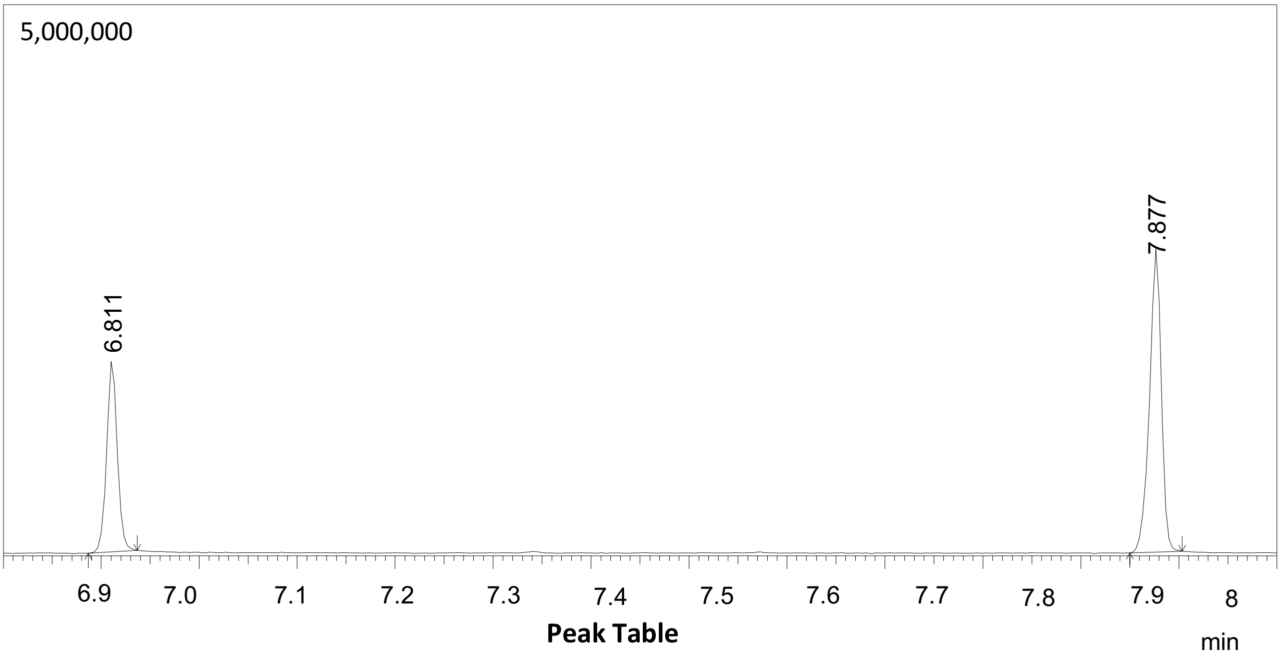


**Peak Table**

| Peak # | Compound | Rt (min) | Area |
|---|---|---|---|
| 1 | 4-CP | 6.811 | 1327675 |
| 2 | MPS | 7.877 | 2323094 |

**b)**

TIC

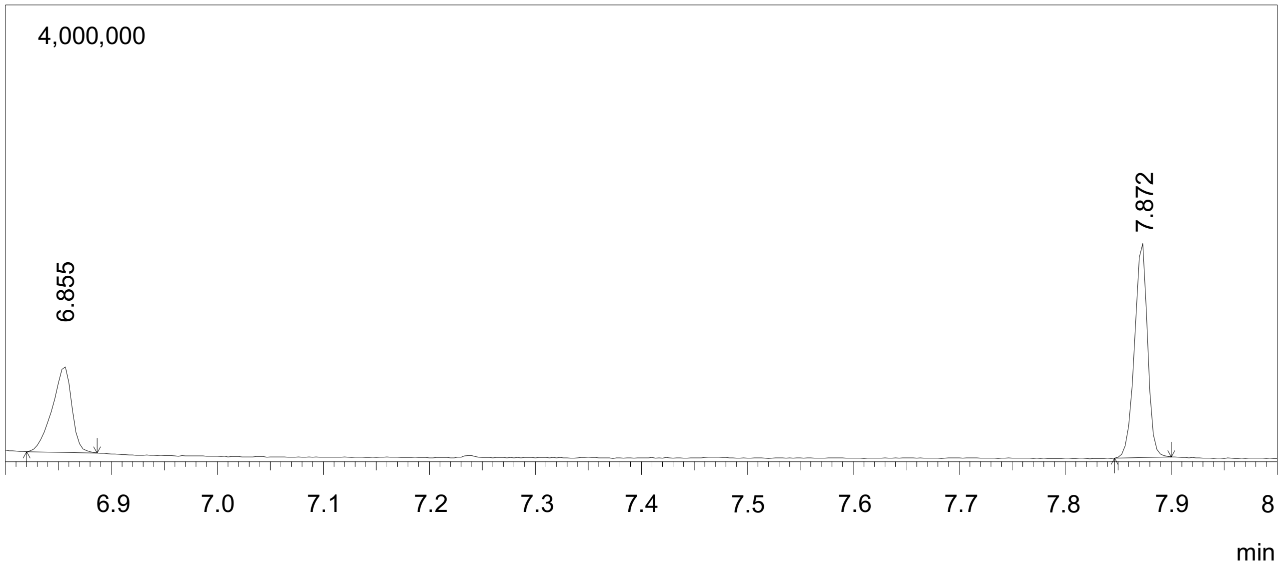


**Peak Table**

| Peak # | Compound | Rt (min) | Area |
|---|---|---|---|
| 1 | 4-CP | 6.855 | 971181 |
| 2 | MPS | 7.872 | 1618738 |

**Figure S14**. GC-MS analysis of the FeMC6*a-catalyzed 4-CP oxidation (a) before and (b) 30 min after sequential $H_2O_2$ and DMPO additions. *Reaction conditions*: [Fe-MC6*a] = 1.0 x $10^{-3}$ mM; [4-CP] = 2.5 mM; [$H_2O_2$] = 2.5 mM; [MPS] = 2.5 mM; [DMPO] = 125 mM; phosphate buffer (50 mM) at pH 6.5 with 50% TFE (v/v).
